# Acute Generalized Exanthematous Pustulosis Secondary to Low-Dose Olanzapine: A Case Report and Mini-Review of the Literature

**DOI:** 10.1192/j.eurpsy.2025.2084

**Published:** 2025-08-26

**Authors:** S. Kukurt

**Affiliations:** 1 Psychiatry, Bezmialem Vakif University, Istanbul, Türkiye

## Abstract

**Introduction:**

Acute generalised exanthematous pustulosis (AGEP) is a rare but severe cutaneous reaction often triggered by medications like antibiotics, calcium channel blockers, and antipsychotics. It is characterised by the rapid onset of non-follicular sterile pustules, fever, and leukocytosis with neutrophilia and eosinophilia. The European SCAR study reports an incidence of 1–5 cases per million annually, with a mortality rate of up to 5% in severe cases (Szatkowski et al. JCM 2022; 11:397). While AGEP is well-documented with various drugs, AGEP induced by low-dose olanzapine is particularly noteworthy due to its rarity and the lack of substantial evidence in existing literature.

**Objectives:**

The aim of this report is to highlight the potential for AGEP to develop even at low doses of olanzapine and to review similar cases from the literature.

**Methods:**

We present the case of a 19-year-old male treated with low-dose olanzapine (2.5 mg) for severe insomnia who developed AGEP. A literature review was conducted to identify other instances of AGEP related to olanzapine. The review utilised PubMed and Google Scholar databases, focussing on cases with detailed patient information including age, gender, dosage, and time to symptom onset (Table 1).

**Results:**

A 19-year-old male with a one-year history of severe sleep deprivation, reporting only one hour of sleep per day in the past week, was prescribed olanzapine 2.5 mg after failed attempts with melatonin, hydroxyzine, and mirtazapine. Two days later, he developed a pruritic rash on the neck, spreading to the trunk and limbs, along with a fever of 38.5°C. Blood tests revealed leukocytosis (12,000/μL), neutrophilia, and eosinophilia but normal liver and kidney function. Dermatological evaluation confirmed AGEP. Olanzapine was discontinued, and cetirizine, an H1 antagonist, was administered. Symptoms improved within 24 hours, and the rash resolved within a week.

**
Table 1: Summary of Olanzapine-Induced AGEP Cases**

**Table:** 

Study	Age	Gender	Dosage (mg)	Symptom onset (days)
Christen et al. Acta Medica (Hradec Kralove) 2006; 49:75-6.	56	Male	10	5
Patel et al. Crit Care Med 2018; 46:469.	38	Male	10	3
Jakhar et al. Indian J Psychiatry 2021; 63:411-3.	57	Female	20	2
OUR CASE	19	Male	2.5	2

**Conclusions:**

This case underscores the need to recognize rare hypersensitivity reactions like AGEP, even at low doses of olanzapine. Early detection and discontinuation of the drug are essential to avoid complications. Literature shows AGEP can occur across various dosages, with symptom onset typically within days. Clinicians should be cautious when prescribing olanzapine, regardless of dose, and closely monitor patients for signs of AGEP to prevent severe outcomes.

**Disclosure of Interest:**

None Declared

